# Differential expression of CD11c defines two types of tissue-resident macrophages with different origins in steady-state salivary glands

**DOI:** 10.1038/s41598-022-04941-5

**Published:** 2022-01-18

**Authors:** Lu Lu, Toshinobu Kuroishi, Yukinori Tanaka, Mutsumi Furukawa, Tomonori Nochi, Shunji Sugawara

**Affiliations:** 1 Division of Oral Immunology, Department of Ecological Dentistry, Tohoku University Graduate School of Dentistry, Sendai, 980-8575 Japan; 2 Department of Dental Anesthesiology and Pain Management, Tohoku University Hospital, Sendai, 980-8574 Japan; 3 International Education and Research Center for Food and Agricultural Immunology, Graduate School of Agricultural Science, Tohoku University, Sendai, 980-8572 Japan

**Keywords:** Cell biology, Immunology

## Abstract

Gland macrophages are primed for gland development and functions through interactions within their niche. However, the phenotype, ontogeny, and function of steady-state salivary gland (SG) macrophages remain unclear. We herein identified CD11c^+^ and CD11c^−^ subsets among CD64^+^ macrophages in steady-state murine SGs. CD11c^−^ macrophages were predominant in the SGs of embryonic and newborn mice and decreased with advancing age. CD11c^+^ macrophages were rarely detected in the embryonic period, but rapidly expanded after birth. CD11c^+^, but not CD11c^−^, macrophage numbers decreased in mice treated with a CCR2 antagonist, suggesting that CD11c^+^ macrophages accumulate from bone marrow-derived progenitors in a CCR2-dependent manner, whereas CD11c^−^ macrophages were derived from embryonic progenitors in SGs. CD11c^+^ and CD11c^−^ macrophages strongly expressed colony-stimulating factor (CSF)-1 receptor, the injection of an anti-CSF-1 receptor blocking antibody markedly reduced both subsets, and SGs strongly expressed CSF-1, indicating the dependency of SG resident macrophage development on CSF-1. The phagocytic activity of SG macrophages was extremely weak; however, the gene expression profile of SG macrophages indicated that SG macrophages regulate gland development and functions in SGs. These results suggest that SG CD11c^+^ and CD11c^−^ macrophages are developed and instructed to perform SG-specific functions in steady-state SGs.

## Introduction

Macrophages, monocytes, and dendritic cells (DCs) are members of the mononuclear phagocyte system, which exhibits multifunctional immune responses, and tissue-resident macrophages have a wide variety of functions in mammalian tissues^1–3^. Recent findings indicated that gland macrophages contribute to gland development and functions by regulating stem cell activation, epithelial cell proliferation, hormone synthesis, and secretion through interactions within their niche^4,5^.

The majority of tissue-resident macrophages are derived from embryonic progenitors and maintained by self-renewal throughout their lifespan. At least three pathways have been implicated in macrophage development^1–3^. Primitive hematopoiesis in the yolk sac gives rise to primitive macrophages without passing through classical monocytic intermediates^6–8^. Erythro-myeloid progenitors (EMPs) generated in the yolk sac migrate to the fetal liver and generate fetal monocytes^9^. Primitive macrophages seed every tissue and give rise to microglia in the brain, while EMP-derived fetal monocytes infiltrate every other tissue and generate the major pool of adult tissue-resident macrophages by diluting the initial primitive macrophage contribution^9^. Hematopoietic stem cell (HSC)-derived monocytes that emerge from the fetal liver contribute to the long-lived macrophage pool at birth while adult hematopoiesis is only starting in the bone marrow (BM)^10^. Adult monocytes contribute to the maintenance of tissue-resident macrophages in some tissues, such as the intestines^1,11,12^.

Salivary glands (SGs) secrete saliva, which includes secretory IgA and other components, into the oral cavity and are important effector sites in the mucosal immune network^13^. In addition to B cells, SGs contain αβ and γδ T cells, NK cells, and macrophages, and SG tissue may function as an antigen-reactive system with the ability to conduct the last stages of a local secretory immune response against invasive pathogens^14^. CD64, the high-affinity IgG receptor FcγRI, and Mer tyrosine kinase (MerTK) were recently identified as core macrophage signature markers^15,16^, and by using CD64 as a macrophage marker, we identified CD64^−^CD11c^+^ classical DCs (cDCs) as well as CD64^+^ macrophages among CD45^+^MHC class II (MHCII)^+^ antigen-presenting cells (APCs) in steady-state murine SGs^17^. SG cDCs were divided into CD103^+^CD11b^−^ type 1 cDCs and CD11c^−^CD11b^+^ type 2 cDCs. Both cDC subsets in SGs markedly expanded in response to the Flt3 ligand, a growth factor of DCs, were replenished by BM-derived precursors, and differentiated from common DC precursors. Furthermore, CD103^+^CD11b^−^ type 1 cDCs possessed an antigen cross-presenting capacity, suggesting that SG cDCs play an important role in maintaining immune homeostasis in SGs. On the other hand, sublingual cDCs preferentially induced regulatory T cells^18,19^. In the course of these studies, we found that CD64^+^ macrophages in SGs comprised CD11c^+^ and CD11c^−^ subsets^17^.

Some characteristics of macrophages in diseased SGs, such as Sjögren’s syndrome, have been described^20^, and we previously identified Th17 cells in the SGs of patients with Sjögren’s syndrome^21^. However, the phenotype, ontogeny and function of steady-state SG macrophages remain unclear. Therefore, in the present study, we aimed to elucidate the ontogeny and function of SG CD11c^+^ and CD11c^−^ steady-state macrophages in mice.

## Results

### Phenotype and localization of macrophage subsets in steady-state SGs

**Figure 1:**
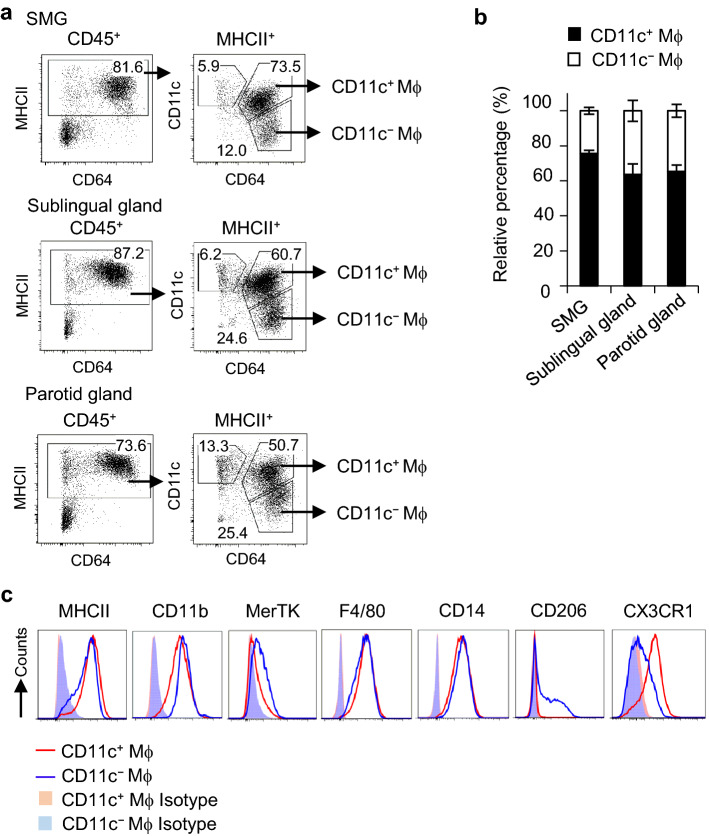
Phenotypes of macrophage subsets in steady-state SGs. Female C57BL/6N mice at 7–12 weeks old were used. (**a**) Gating strategy to identify macrophage subsets in steady-state SMGs, and sublingual and parotid glands by flow cytometry. Representative plots are shown. (**b**) Relative percentages of CD11c^+^ and CD11c^−^ macrophages in SMGs and the sublingual and parotid glands. Bars represent the mean ± SD of three mice per group. (**c**) Histograms represent the expression of surface markers. Red and blue lines represent the specific staining of CD11c^+^ and CD11c^−^ macrophages, respectively, while shaded histograms represent the unstained control. Results are representative of three independent experiments. *Mϕ* macrophage.

Cells of the submandibular glands (SMGs) from wild-type mice were isolated by enzymatic digestion using collagenase and analyzed by flow cytometry. By combining the expression of CD64 and CD11c, CD45^+^MHCII^+^ APCs in SMGs were separated into CD64^+^ macrophages and CD64^−^CD11c^+^ cDCs (Fig. 1a). CD64^+^ macrophages were further classified into the CD11c^+^ and CD11c^−^ subsets. Approximately 90% of APCs in SMGs were macrophages, and CD11c^+^ macrophages were predominant in the SMGs of adult mice. Since the composition of cDCs and CD11c^+^ and CD11c^−^ macrophages in the sublingual and parotid glands was similar to that in SMGs (Fig. 1a,b), we mainly examined SMGs as representative SGs in subsequent experiments.

CD11c^+^ and CD11c^−^ macrophages equally expressed MHCII, CD11b, the macrophage marker F4/80, and CD14 (Fig. 1c). CD11c^−^ macrophages also more strongly expressed MerTK and the mannose receptor CD206 than CD11c^+^ macrophages, while CX3CR1 was strongly expressed in CD11c^+^ macrophages. These results indicated that macrophages exist as predominant APCs in the steady-state SGs of adult mice and that SG macrophages comprise CD11c^+^ and CD11c^−^ subsets, which may have different ontogenies and functions.

**Figure 2:**
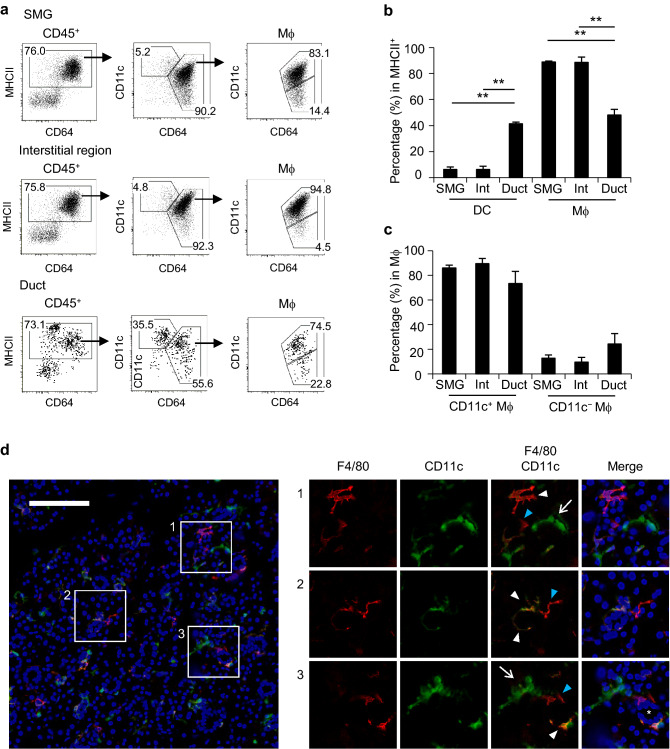
Localization of macrophage subsets in steady-state SGs. Female C57BL/6N mice at 7–12 weeks old were used. (**a**–**c**) DCs and macrophages in whole SMGs and in the interstitial region and purified ducts of SMGs were analyzed by flow cytometry. Representative FACS plots (**a**), the percentages of DCs and macrophages in MHCII^+^ cells (**b**), and percentages of macrophage subsets (**c**) are shown. Bars represent the mean ± SD (*n* = 3). **P < 0.01 by the Student’s *t* test with Welch’s correction. (**d**) Sections from SMG were stained with anti-F4/80 (red) and anti-CD11c (green). Images merged with DAPI-counterstained images are shown on the right. Scale bar: 100 μm. White arrowhead: F4/80^+^CD11c^+^. Blue arrowhead: F4/80^+^CD11c^-^. White arrow: F4/80^-^CD11c^+^. *Duct. Data were representative of three individual experiments.

To clarify the localization of cDCs and macrophages in SGs, we examined their frequencies in whole SMG, enriched ductal, and remaining interstitial fractions. Most macrophages existed in the interstitial region, while cDCs were enriched by approximately sevenfold in the ductal region (Fig. 2a,b). CD11c^+^ macrophages were predominant in the ductal and interstitial regions; however, the frequency of CD11c^−^ macrophages slightly increased in the ductal region (Fig. 2a,c), indicating that most CD11c^+^ and CD11c^−^ macrophages reside in the interstitial and ductal regions and also that cDCs and some CD11c^−^ macrophages are potently associated with the ductal region. In support of these results, F4/80^+^CD11c^−^ and F4/80^+^CD11c^+^ macrophages as well as F4/80^−^CD11c^+^ cDCs were detected in the ductal and stromal regions by immunohistochemistry (Fig. 2d). Immunohistochemistry also revealed no significant differences in the morphologies of CD11c^+^ and CD11c^−^ macrophages.

### Age-dependent transition of macrophage subsets in steady-state SMGs

**Figure 3:**
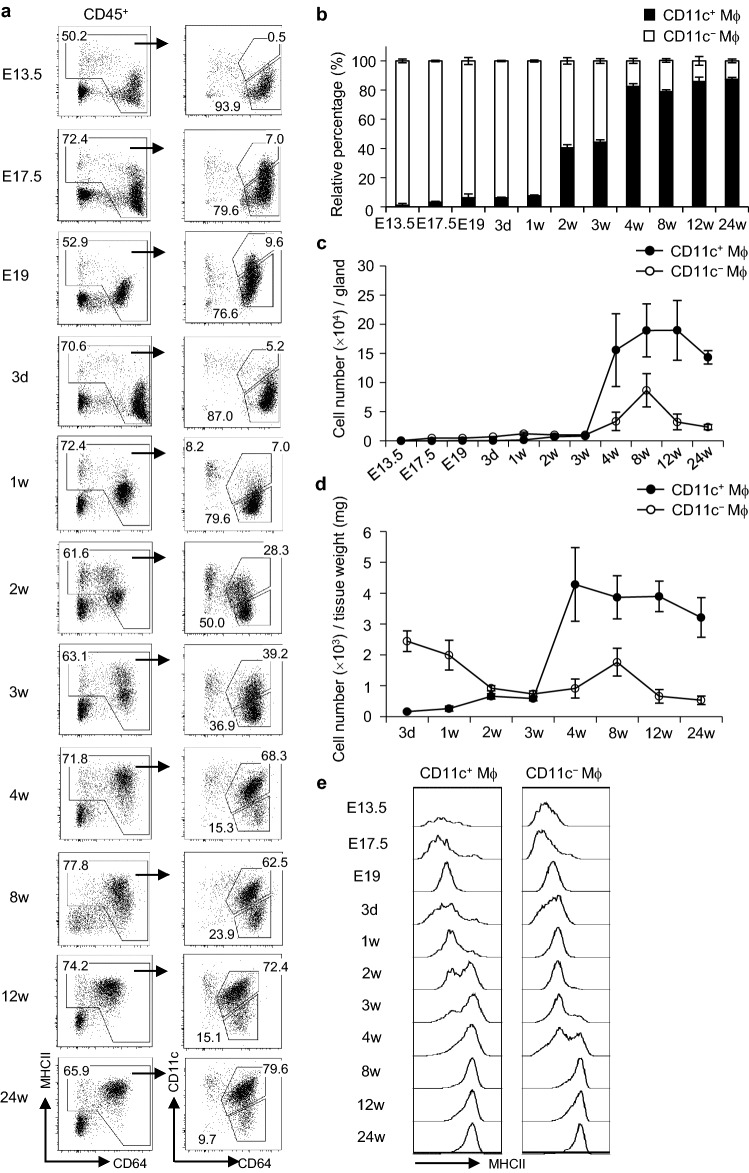
Age-dependent transition of macrophage subsets in steady-state SMGs. (**a**–**e**) Macrophage subsets in steady-state SMGs from mice at E13.5, E17.5, and E19 (embryo) and at 3 days (newborn) and 1, 2, 3, 4, 8, 12, and 24 weeks after birth were analyzed by flow cytometry. Representative FACS plots (**a**), the proportion of CD11c^+^ and CD11c^−^ macrophages (**b**), cell numbers per gland (**c**), cell numbers per tissue weight (**d**), and representative histograms for the expression of MHCII (**e**) are shown. Bars represent the mean ± SD (*n* = 3). Results are representative of three independent experiments. *Mϕ* macrophage.

To examine the ontogeny of SG macrophage subsets, we analyzed macrophages in steady-state SMGs from mice at E13.5, E17.5, and E19 (embryo) and at 3 days (newborn) and 1, 2, 3, 4, 8, 12, and 24 weeks after birth by flow cytometry. The results obtained showed that CD11c^−^ macrophages were a major population in the SMGs of the embryonic period and newborn mice, and then decreased with advancing age after birth (Fig. 3a). Inversely, CD11c^+^ macrophages were rarely detected in the SMGs of the embryonic period and newborn mice, and then rapidly expanded with advancing age. The proportion of CD11c^+^ macrophages in young (4 weeks old) mice was similar to that in adult (8–24 weeks old) mice (Fig. 3b). The cell numbers of CD11c^+^ and CD11c^−^ macrophages both rapidly expanded concomitantly with the growth of SMGs at 4 weeks (Fig. 3c); however, the number of CD11c^−^ macrophages per tissue weight slightly decreased after birth (Fig. 3d). In addition, the expression of MHCII in CD11c^+^ and CD11c^−^ macrophages increased 2 and 4 weeks after birth, respectively (Fig. 3e). The number of CD11c^+^ cDCs also slightly increased at 2–4 weeks (Supplementary Fig. S1). Therefore, marked changes in the composition of macrophage subsets and their maturation status occurred with SG development.

### HSC dependence of SMG CD11c^+^ macrophages

**Figure 4:**
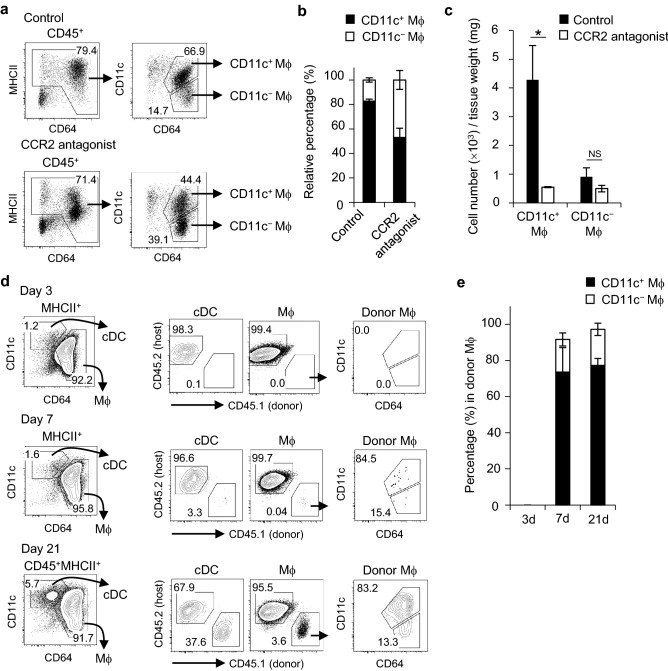
HSC dependency of macrophage subsets in steady-state SMGs. (**a**–**c**) Macrophage subsets in steady-state SMGs from control or CCR2 antagonist-treated mice. Newborn mice were administered the CCR2 antagonist from postnatal day 3 for 4 weeks, and SMG tissues were then harvested. Representative FACS plots (**a**). Data are representative of three independent experiments. The proportion (**b**) and absolute numbers (**c**) of CD11c^+^ and CD11c^−^ macrophages in steady-state SMGs from control or CCR2 antagonist-treated mice. (**d**,**e**) Sublethally irradiated female CD45.2^+^ mice (7 weeks old) mice were adoptively transferred with CD45.1^+^ BM cells. SMG tissues were harvested at the indicated time points and the percentage of donor-derived (CD45.1^+^) cDCs and macrophage subsets were analyzed by flow cytometry. Representative FACS plots (**d**) and the percentages of the CD11c^+^ and CD11c^−^ subsets in donor macrophages (**e**) are shown. Bars represent the mean ± SD (*n* = 3). *P < 0.05 by the Student’s *t* test with Welch’s correction. Results are representative of three independent experiments. *Mϕ* macrophage, *NS* not significant.

The result showing that CD11c^+^ macrophages expanded in SMGs after birth suggested that CD11c^+^ macrophages differentiated from BM-derived monocytes. Since the emigration of monocytes from BM requires signals mediated by the chemokine receptor CCR2^22^, we examined the effects of a CCR2 antagonist on the accumulation of macrophages in steady-state SMGs. The treatment of mice with a CCR2 antagonist reduced the percentage of CD11c^+^ macrophages (Fig. 4a,b). The number of CD11c^+^ macrophages was also significantly reduced by the treatment, whereas that of CD11c^−^ macrophages was not (Fig. 4c). Furthermore, 21 days after CD45.1^+^ BM cell transplantation into sublethal irradiated CD45.2^+^ mice, differentiated donor-derived macrophages were mainly the CD11c^+^ phenotype (Fig. 4d,e); however, a small number of CD11c^−^ macrophages was detected 7 and 21 days after the BM transplantation. These results suggest that CD11c^+^ macrophages mainly accumulated from HSC-derived progenitors in a CCR2-dependent manner, while CD11c^−^ macrophages were mostly derived from embryonic progenitors and self-renewed in SGs.

### Colony-stimulating factor (CSF)-1-dependent accumulation of SMG macrophages

**Figure 5:**
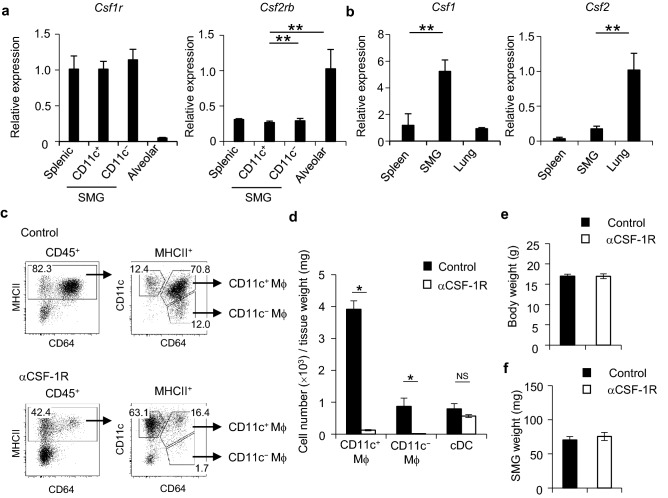
CSF-1 dependency of macrophage subsets in steady-state SMGs. (**a**,**b**) Female C57BL/6N mice at 8 weeks old were used. The mRNA expression levels of *Csf1r* and *Csf2rb* were analyzed by quantitative RT-PCR (**a**). Splenic and alveolar macrophages were the positive controls for *Csf1r* and *Csf2rb*, respectively. Expression levels were normalized to *β-actin* levels and expressed as relative expression based on the value of splenic macrophages (*Csf1r*) or alveolar macrophages (*Csf2rb*). mRNA expression levels of *Csf1* and *Csf2* in spleen, SMG, and lung tissues (**b**). The spleen and lungs were positive controls for *Csf1* and *Csf2*, respectively. Expression levels were normalized to *β-actin* levels and expressed as relative expression based on the value of the spleen (*Csf1*) or lungs (*Csf2*). Results are representative of three independent experiments. (**c**–**f**) Macrophage subsets in steady-state SMGs from control or anti-CSF-1R mAb-injected mice. Female C57BL/6N mice at 7 weeks old were injected with anti-CSF-1R mAb on days 0, 1, and 2, and then analyzed on day 5. Representative FACS plots (**c**), the absolute numbers of CD11c^+^ and CD11c^−^ macrophages (**d**), body weight (**e**), and SMG weight (**f**) in steady-state SMGs from control or anti-CSF-1R-injected mice are shown. Results are representative of two independent experiments. Bars represent the mean ± SD (*n* = 3). **P < 0.01, *P < 0.05 by the Student’s *t* test with Welch’s correction. *Mϕ* macrophage, *NS* not significant.

CSF-1 or CSF-2 is indispensable for the development and homeostasis of tissue-resident macrophages. CSF-1 is produced in the spleen and capable of maintaining splenic macrophages locally^23^, whereas the production of CSF-2 in the lungs is required for the development of alveolar macrophages^24,25^. To examine whether CSF-1 or CSF-2 influences the development of SG macrophages, SMG CD11c^+^ and CD11c^−^ macrophages and splenic and alveolar macrophages were sorted (Supplementary Fig. S1), and the expression of *Csf1r* and *Csf2rb* mRNA was examined to assess CSF-1 receptor (CSF-1R) and CSF-2R expression in these macrophages. Splenic and alveolar macrophages were used as positive controls for the expression of *Csf1r* and *Csf2rb* mRNA, respectively. The results obtained showed that SMG CD11c^+^ and CD11c^−^ macrophages strongly expressed *Csf1r*, but not *Csf2rb* (Fig. 5a). We also measured CSF-1 and CSF-2 expression levels in SMGs. Homogenates of the spleen and lungs were used as positive controls for the expression of *Csf1* and *Csf2* mRNA, respectively. The expression of *Csf1* was significantly stronger in SMGs than in the spleen, whereas that of *Csf2* was significantly weaker in SMGs than in the lungs (Fig. 5b), suggesting the dependency of SG CD11c^+^ and CD11c^−^ macrophage development on CSF-1. To confirm this, an anti-CSF-1R blocking antibody was intraperitoneally injected. The results obtained showed that the percentages and total cell numbers of SMG CD11c^+^ and CD11c^−^ macrophages markedly decreased (Fig. 5c,d), and the injection of anti-CSF-1R did not influence the number of SMG cDCs (Fig. 5d) or body and SMG weights (Fig. 5e,f). Collectively, these results clearly indicate that the production of CSF-1 in SGs plays a critical role in the maintenance and survival of CSF-1R-expressing SG resident macrophages.

We then investigated whether the early-in-life depletion of SG macrophages affects tissue morphology and development. One-week-old mice were intraperitoneally injected with anti-CSF-1R on days 0, 1, 2, 14, and 28 and examined on day 35. The injection of anti-CSF-1R markedly reduced the numbers of CD11c^+^ and CD11c^−^ macrophages (Supplementary Fig. S2a,b), but did not affect body and SMG weights or the morphology of SMG (Supplementary Fig. S2c–e). These results suggest that, after birth, SG macrophages did not affect the morphology or development of SGs.

### Mastication does not affect the development of SMG macrophages

**Figure 6:**
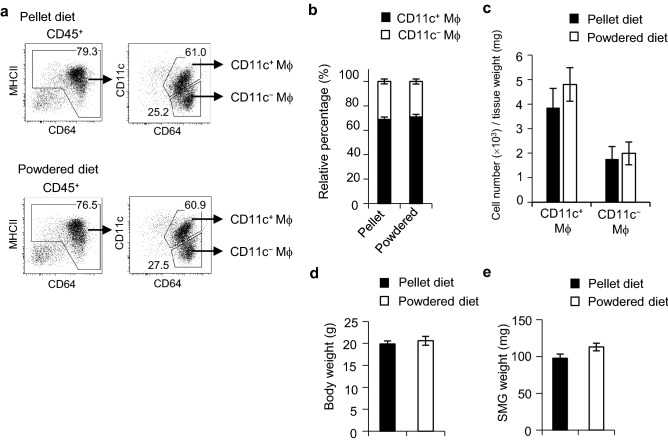
Effects of the diet form on macrophage subsets in steady-state SMGs. One-week-old mice and their mothers were fed standard pellets or a powdered diet. After 8 weeks, SMGs from the pups were analyzed by flow cytometry. Representative FACS plots (**a**), the proportions (**b**) and absolute numbers of CD11c^+^ and CD11c^−^ macrophages (**c**), body weight (**d**), and SMG weight (**e**) are shown. Bars represent the mean ± SD (*n* = 3). Results are representative of three independent experiments. *Mϕ* macrophage.

Since mastication is important for the maintenance of SG functions, such as salivary secretion^26^, and the number of CD11c^+^ macrophages increased in mice aged 2–4 weeks (Fig. 3a,b) when they began to be weaned and eat the standard diet, we investigated whether mastication affected the development of CD11c^+^ macrophages. One-week-old mice and their mothers were fed standard pellets or a powdered diet. After 8 weeks, the proportion of CD11c^+^ and CD11c^−^ macrophages in SMGs was analyzed. No significant differences were observed in the percentage or number of CD11c^+^ macrophages between the pellet and powdered diet groups (Fig. 6a–c). Body and SMG weights were also unchanged between the groups (Fig. 6d,e). These results indicated that mastication did not affect the development of SG resident macrophages.

### Functional perspectives of SG macrophages

**Figure 7:**
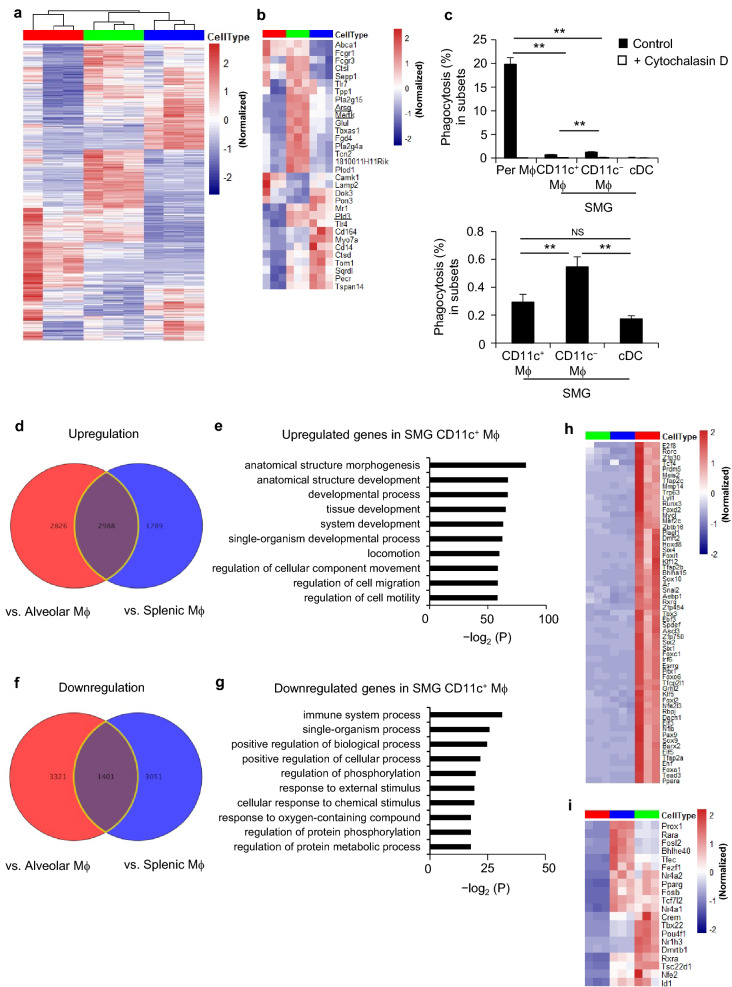
Functional analysis of SMG macrophages. Female C57BL/6N mice at 8 weeks old were used. (**a**,**b**) Heat map of mRNA expression profiles discriminating SMG CD11c^+^ macrophages from alveolar and splenic macrophages (**a**). The gene expression profiles of core macrophage signatures^10^ are shown (**b**). Cell types include SMG CD11c^+^ (red), splenic (green), and alveolar macrophages (blue). (**c**) Phagocytosis was evaluated by an in vitro assay with pHrodo *E. coli* BioParticles in the presence or absence of cytochalasin D (upper panel) and with apoptotic cells (lower panel). Bars represent the mean ± SD. **P < 0.01 by the Student’s *t* test with Welch’s correction. Results are representative of three independent experiments. (**d**,**f**) Venn diagrams showing differentially expressed genes (significantly up- or down-regulated by twofold or more) in SMG CD11c^+^ macrophages relative to alveolar and splenic macrophages. (**e**,**g**) The most significant GO terms for up-regulated (**e**) and down-regulated (**g**) genes in SMG CD11c^+^ macrophages. The top 10 significantly enriched GO terms are shown. (**h**,**i**) Heat maps showing differentially expressed encoding transcription factors (significantly up- or down-regulated by fivefold or more) in SMG CD11c^+^ macrophages (red) relative to splenic (green) and alveolar macrophages (blue). Microarray data were collected from three independent experimental samples for each cell type. *Mϕ* macrophage.

The gene expression profiles of SMG CD11c^+^ macrophages, alveolar macrophages, and splenic macrophages were examined and compared. SMG CD11c^−^ macrophages were not assessed because their number was too small for analysis. Hierarchical clustering demonstrated that SMG CD11c^+^ macrophages did not cluster with splenic and alveolar macrophages (Fig. 7a), suggesting a weak relationship between these macrophages. We then compared the expression of 39 core macrophage signature genes^15^. Normalized relative gene expression demonstrated that SMG CD11c^+^ macrophages expressed lower levels of *Arsg* encoding arylsulfatase G, *Mertk* encoding MerTK, and *Pld3* encoding phospholipase D3 (Fig. 7b). MerTK is a receptor for apoptotic cell uptake^27^, and arylsulfatase G and phospholipase D3 are lysosomal enzymes^28,29^, suggesting that SG macrophages exhibit weak phagocytic activity. An in vitro analysis showed that although SMG CD11c^−^ macrophages phagocytosed BioParticle and apoptotic cells more efficiently than SMG CD11c^+^ macrophages, the phagocytic activities of SMG CD11c^+^ and CD11c^−^ macrophages were markedly weaker than that of peritoneal macrophages (Fig. 7c). Phagocytosis by macrophages was abrogated by the addition of cytochalasin D, the F-actin disrupter^30^, to the culture.

A gene ontology (GO) pathway analysis identified 2988 genes that were up-regulated in SMG CD11c^+^ macrophages (Fig. 7d), which displayed enrichment for pathways involved in developmental processes, such as anatomical structure morphogenesis, anatomical structure development, developmental process, tissue development, system development, and single-organism developmental process, and migration processes including locomotion and the regulation of cellular components, cell migration, and cell motility (Fig. 7e). In contrast, 1401 genes were down-regulated in SMG CD11c^+^ macrophages (Fig. 7f), which displayed enrichment for pathways involved in immune system processes, single organism processes, the positive regulation of biological processes, and the positive regulation of cellular processes (Fig. 7g). In comparisons with alveolar and splenic macrophages, 59 and 20 transcription factors were up- and down-regulated by more than fivefold, respectively, in SMG CD11c^+^ macrophages (Fig. 7h,i). Therefore, the transcription profile is unique to SG macrophages, indicating that they are specifically primed to function in SGs.

**Figure 8:**
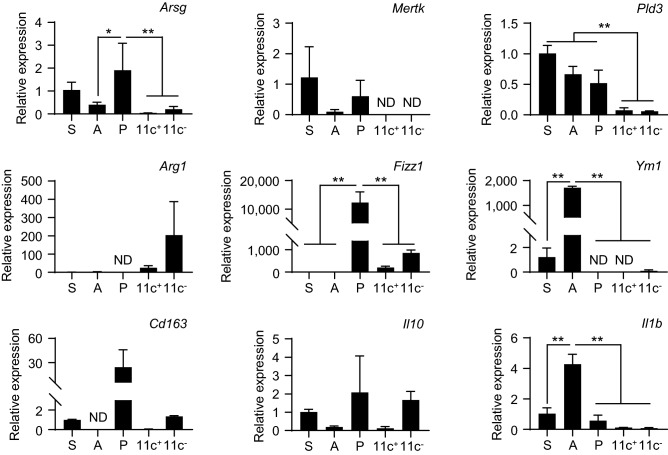
mRNA expression levels of macrophage signature genes in SMG macrophages. Female C57BL/6N mice at 8 weeks old were used. The mRNA expression levels of *Arsg*, *Mertk*, *Pld3*, *Arg1*, *Fizz1*, *Ym1*, *Cd163*, *Il10*, and *Il1b* were analyzed by quantitative RT-PCR. Expression levels were normalized to β-actin levels and expressed as relative expression based on the value of splenic macrophages. Bars represent the mean ± SD (n = 2 or 3). If two or more sets of triplicate samples were lower than detection limit, results were expressed as “not detected (ND)”. *S* splenic macrophages, *A* alveolar macrophages, *P* pancreatic macrophages, *11c*^*+*^ SMG CD11c^+^ macrophages, *11c*^−^ SMG CD11c^−^ macrophages. **P < 0.01, *P < 0.05 by a one-way ANOVA using Tukey’s multiple comparisons tests.

We also examined the mRNA expression levels of macrophage signature genes in SMG CD11c^+^ and CD11c^−^ macrophages. In addition to splenic and alveolar macrophages, macrophages from the exocrine pancreas (pancreatic stroma) were used as an exocrine control tissue. Consistent with the gene expression profiles shown in Fig. 7b, the mRNA expression levels of *Arsg*, *Mertk*, and *Pld3* were markedly lower in SMG CD11c^+^ and CD11c^−^ macrophages than in splenic and pancreatic macrophages (Fig. 8). We then assessed canonical pro- (*Il1b*) and anti-inflammatory gene expression (*Arg1*, *Fizz1*, *Cd163*, *Ym1*, and *Il10*)^31^. SMG CD11c^−^ macrophages expressed *Arg1*, *Fizz1*, *Cd163*, *Ym1*, and *Il10*, and the expression of *Il1b* was similar between SMG CD11c^+^ and CD11c^−^ macrophages. Exocrine pancreatic macrophages highly expressed *Fizz1* and *Cd163*, while alveolar macrophages highly expressed *Ym1*. These results suggest that CD11c^−^ macrophages exhibit immunoregulatory functions in SGs.

## Discussion

The present study showed that CD64^+^ macrophages among CD45^+^MHCII^+^ APCs in murine SGs were phenotypically divided into CD11c^+^ and CD11c^−^ subsets. CD11c^−^ macrophages dominantly existed in the SGs of the embryonic period and neonatal mice and decreased after birth. In contrast, CD11c^+^ macrophages appeared after birth in SGs and rapidly expanded with advancing age. Since CD11c^+^ alveolar macrophages develop from CD11c^−^ fetal monocytes in the lungs^19^, SG CD11c^−^ macrophages may give rise to CD11c^+^ macrophages after birth. However, the present study showed that CD11c^+^ macrophages expanded in a CCR2-dependent manner and differentiated from BM-derived progenitors, while CD11c^−^ macrophages did not change in number following a treatment with a CCR2 antagonist and only weakly differentiated from transferred BM cells. A mass cytometric analysis suggested that fetal-derived tissue-resident macrophages in the mammary gland are CD206^Hi^ cells^32^, and SG CD11c^−^ macrophages preferentially expressed CD206 (Fig. 1c). Therefore, it is conceivable that SG CD11c^−^ macrophages are mainly derived from fetal progenitors and self-renewed in SGs, while CD11c^+^ macrophages differentiated from BM-derived progenitors after birth in SGs.

The present results demonstrated that macrophages in embryo SMGs were CD11c^−^MHCII^−^, which is consistent with previous findings showing that embryo-derived cardiac macrophages present at birth were CX3CR1^+^MHCII^−^^33^. This study also revealed that the expression of MHCII in SG macrophages started to increase after birth. The expression of MHCII is fine-tuned to function in various parameters, including developmental stages, the activation status, and exposure to extracellular stimuli, and MHCII loads antigenic peptides and guides the development and activation of antigen-specific CD4^+^ T cells^34,35^. Therefore, it is conceivable that SG macrophages acquire the ability of specific antigen presentation after birth.

The present study showed that most CD11c^+^ and CD11c^−^ macrophages resided in interstitial and ductal regions and that cDCs and some CD11c^−^ macrophages were strongly associated with the ductal region. The result may not be consistent with recent findings showing that CD11c^+^ macrophages were associated with acini and ducts in SGs using the CD11c-yellow fluorescent protein (YFP) reporter strain^36^. We attributed this discrepancy to differences in the gating strategy. We initially separated CD45^+^MHCII^+^ APCs in SGs into CD64^+^ macrophages and CD64^−^CD11c^+^ cDCs while they gated CD11c-YFP, which excluded CD11c^−^ macrophages, and then analyzed the expression of CD64.

The present study showed that the development of SG CD11c^+^ and CD11c^−^ macrophages depended on CSF-1R signaling, which supports CSF-1 being released from the serous acinar cells of SGs to function in the survival, recruitment, and proliferation of SG macrophages^4,5,37,38^. The results obtained also showed that SG CD11c^+^ macrophages expanded rapidly in mice aged 2–4 weeks when they began to be weaned and eat the standard diet. Since mechanical stress has been suggested to explain the replacement of embryo-derived macrophages by circulating monocyte-derived macrophages^12,33^, we examined whether mastication influenced the development of SG CD11c^+^ macrophages; however, the powdered diet did not change the proportion of SG resident macrophages. Previous studies also demonstrated that intestinal commensal microbiota controlled macrophage turnover^11,12^, therefore, oral commensal microbiota may promote SG CD11c^+^ macrophage development. In the intestines, TGF-β was identified as indispensable for the differentiation of monocytes into macrophages^39^. Therefore, further studies are needed to identify the SG microenvironmental factors that control macrophage development in order to maintain SG homeostasis.

In mammary glands, which are exocrine glands similar to SGs, macrophages distribute during distinct phases of development and remodeling^40^, and ductal macrophages survey the mammary epithelium and facilitate tissue remodeling^41^, indicating that mammary gland macrophages play an essential role in gland development. However, there is still no clear evidence to show that macrophages contribute to SG development. In mouse SMG morphogenesis, the gland is highly branched by E14, and functional differentiation begins at E15 and continues to birth^42^. The present study showed that CD11c^−^ macrophages were already detected at E13.5 (Fig. 3) and potently associated with the ductal region (Fig. 2). Therefore, CD11c^−^ macrophages may play an essential role in gland morphogenesis in association with the ductal region in fetal SGs. The treatment of SMGs isolated from E12.5 mouse embryos with anti-CSF-1 or anti-CSF-1R reduced the numbers of F4/80^+^ macrophages and suppressed the development of SMGs; however, the latter effect may be explained by macrophage-independent pathways^43^. The specific depletion of macrophages by novel approaches, such as the use of CD64-diphtheria toxin receptor mice, may contribute to a more detailed understanding of the exact role of macrophages in SG morphogenesis.

In conclusion, the present results indicate that fetal macrophages are present in SGs as early as E13.5 for the development of SGs, and that SG macrophages continues to function after birth in steady-state SGs by inducing proliferation of fetal-derived macrophages and receiving supplies from BM-derived progenitors. CD11c is a key marker that distinguishes between BM- and embryonic-derived SG macrophages. The present study showed that the phagocytic activity of SG macrophages was extremely weak; however, the gene expression profile of SMG macrophages suggested that SG macrophages regulate gland development and functions in SGs. This view is partly supported by recent findings indicating that murine SG macrophages allow the patrolling of tissue by tissue-resident memory CD8^+^ T cells for homeostatic organ surveillance^36^, and attenuate radiotherapy-induced dry mouth through the activation of the Hedgehog pathway^44^. The methods employed in the present study do not provide the same level of evidence as other experiments, such as lineage tracing protocols or parabiosis. Therefore, the further characterization of SG macrophages is important for evaluating the contribution of SG macrophage subpopulations to SG homeostasis and to the pathology of SG dysfunctions and their therapeutic purposes.

## Materials and methods

### Mice

CD45.2^+^ C57BL/6N mice were purchased from CLEA Japan (Tokyo, Japan). Male and female mice were placed into the same cage at 8 p.m. Mice were separated the next morning and regarded as embryonic development day 0.5 (E0.5). Congenic CD45.1^+^ C57BL/6 mice were provided by RIKEN BRC (Tsukuba, Japan). All experiments were approved by the Institutional Animal Care and Use Committee of Tohoku University (approved number: 2017DnA-006) and were performed in accordance with their guidelines and regulations. This study was carried out in compliance with the ARRIVE Essential 10 Guidelines.

### Preparation of single-cell suspensions

Mice were euthanized with isoflurane and then perfused with 20 ml of phosphate-buffered saline (PBS) through the left ventricle to remove circulating blood. SG cells were purified following previously described protocols^17^, and subjected to a flow cytometric analysis. To enrich ducts, SMGs were digested with collagenase, hyaluronidase, and DNase for 1 h and pipetted with a 25-ml pipette. Ducts were manually selected using a dissection microscope and processed to single cells as described above. The spleen and lungs were cut into small pieces. Spleen pieces were incubated with 1 mg/ml of collagenase D (Roche, Basel, Switzerland) and 0.1 mg/ml of DNase I in RPMI 1640 medium containing 10% fetal bovine serum (FBS) at 37 °C for 30 min with shaking. Spleen cells were depleted of red blood cells by hypotonic lysis. Lung pieces were incubated with 0.4 mg/ml of collagenase type IV (Sigma-Aldrich, St Louis, MO, USA) in RPMI 1640 medium containing 10% FBS at 37 °C for 45 min with shaking. Resident peritoneal macrophages were isolated by an injection of 5 ml of ice-cold PBS into the peritoneal cavity. Pancreatic cells were isolated according to previously described protocols^45^. Briefly, the retrograde infusion of 1 ml of 1 mg/ml collagenase type IV in Hanks’ balanced salt solution (HBSS) into the pancreatic duct was performed. The inflated pancreas was then removed and digested in HBSS at 37 °C for 8.5 min with gentle shaking. After washing with HBSS, the cell suspension was filtered using a 70-μm cell strainer to remove the islets.

### BM chimera

BM cells from congenic CD45.1^+^ C57BL/6 were collected as previously described^17^. CD45.2^+^ recipient mice were sublethally γ-irradiated with a single dose of 5 Gy and intravenously injected with 2 × 10^6^ CD45.1^+^ total BM cells. Chimeric mice were analyzed 3, 7, and 21 days after BM transplantation.

### Flow cytometry

Flow cytometry was performed as previously described^17^. Cells were treated with an anti-CD16/32 antibody (2.4G2; produced in-house) to block Fc receptors and then stained with antibodies. The antibodies used were listed in Supplementary Table S1. Dead cells were excluded using DAPI (Dojindo, Kumamoto, Japan). Data were acquired on an LSRFortessa cell analyzer (BD Biosciences, San Jose, CA, USA) and analyzed using FlowJo software (Tree Star, Ashland, OR, USA). Absolute cell numbers were measured by CountBright absolute counting beads (Life Technologies, Carlsbad, CA, USA). Cell sorting was performed using a FACSAria II cell sorter (BD Biosciences). Splenic macrophages were sorted as live gated CD45^+^B220^−^MHCII^+^F4/80^+^CD64^+^CD11c^int^ cells. Alveolar macrophages were sorted as live gated CD45^+^MHCII^low^Siglec-F^+^CD64^+^F4/80^+^CD11c^+^CD11b^−^ cells. Macrophages from the exocrine pancreas (pancreatic stroma) were sorted as live gated CD45^+^B220^-^MHCII^+^CD64^+^F4/80^+^ cells (Supplementary Fig. S3).

### Immunohistochemistry

Tissues were fixed in 4% (w/v) paraformaldehyde at 4 °C overnight and embedded in O.C.T. compound (Sakura Finetek, Tokyo, Japan). To analyze the localization of macrophages, immunohistochemistry was performed on frozen tissue sections (10 μm). Specifically, sections were treated with REAL Target Retrieval Solution (DAKO, Carpinteria, CA, USA) at 98 °C for 40 min to retrieve the antigen and incubated in 3% H_2_O_2_ at room temperature (RT) for 10 min to inhibit endogenous peroxidase. After washing, sections were incubated with 0.5% (w/v) blocking reagent (PerkinElmer, Waltham, MA, USA) at RT for 30 min to block the non-specific binding of antibodies and then treated with primary antibodies at 4 °C overnight followed by secondary antibodies at RT for 1 h. The primary antibodies used in the present study were rabbit anti-CD11c (clone D1V9Y, 1:100, Cell Signaling Technology, Danvers, MA, USA) and rat anti-F4/80 (clone BM8, 1:50, BioLegend). Secondary antibodies were horseradish peroxidase (HRP)-conjugated donkey anti-rabbit IgG (1:2000, Jackson ImmunoResearch, West Grove, PA, USA) and Alexa Fluor 647-conjugated donkey anti-rat IgG (1:200, Jackson ImmunoResearch). The enzyme activity of HRP was visualized using the TSA Plus fluorescein system (PerkinElmer). Sections were then counterstained with DAPI. Tissue images were obtained using a BZ-9000 microscope (Keyence, Osaka Japan).

### Quantitative real-time PCR

Total RNA from tissues and sorted macrophages was extracted by the RNeasy Mini Kit (QIAGEN, Hilden, Germany). Complement DNA (cDNA) was synthesized using a Transcriptor First Strand cDNA Synthesis Kit (Roche). Quantitative RT-PCR was performed using the SYBR Select Master Mix and StepOnePlus real-time PCR system (Applied Biosystems, Waltham, MA, USA). The primers used for quantitative RT-PCR are shown in Supplementary Table S2. Gene expression was normalized to *β-actin* mRNA levels.

### CCR2 antagonist treatment in vivo

Newborn mice were administered 50 mg/kg of the CCR2 antagonist propagermanium (Sigma-Aldrich) via daily oral gavage and 0.1 mg/ml propagermanium in drinking water from postnatal day 3 for 4 weeks. The vehicle control group was given an equal volume of tap water. Body and SMG weights were measured and SMG cells were subjected to flow cytometry.

### Anti-CSF-1R treatment in vivo

Anti-CSF-1R mAb was purified from the ascites of nude mice injected with AFS98 hybridoma cells (Riken BRC, Japan). C57BL/6 female mice were injected with 2, 0.5, and 0.5 mg of anti-CSF-1R mAb in 300 μl sterile PBS by an intraperitoneal injection on days 0, 1, and 2, respectively, and then analyzed on day 5. To examine the effects of the early-in-life depletion of SG macrophages, one-week-old mice were intraperitoneally injected with 0.1, 0.025, 0.025, 0.025, and 0.025 mg/g body weight of anti-CSF-1R mAb in 10 μl/g body weight sterile PBS on days 0, 1, 2, 14, and 28, respectively, and then analyzed on day 35.

### Assessment of phagocytosis in vitro

SMG or peritoneal cells were stained with anti-CD45 (clone 30-F11)-APC (BioLegend, San Diego, CA, USA), and then purified using the EasySep Mouse APC Selection Kit (STEMCELL Technologies, Canada). The pHrodo™ Green *Escherichia coli* BioParticles conjugate for phagocytosis (Thermo Fisher Scientific) was used as indicated by the manufacturer’s protocol. One hundred microliters of the pHrodo™ Green *E. coli* BioParticle suspension and purified SMG or peritoneal CD45^+^ cells (1 × 10^6^) were cocultured at 37 °C for 1 h. To inhibit phagocytosis, 5 μg/ml cytochalasin D (Wako Pure Chemical, Osaka, Japan) was added to culture media for 45 min as a negative control. Thymocytes were stained with the CellTrace Far Red Cell Proliferation kit (Thermo Fisher Scientific) and then incubated with 1 μM dexamethasone at 37 °C for 6 h to induce apoptosis. Apoptotic cells and SMG CD45^+^ cells (1 × 10^6^) were co-cultured at a 1:1 ratio at 37 °C for 3 h. Cells were further analyzed by flow cytometry after the incubation.

### Microarray analysis

Macrophage subsets were sorted from the SMG, lung, and splenic single-cell suspensions of C57BL/6N mice using the FACS Aria II cell sorter (BD Biosciences). Gating strategies are shown in Supporting Information Fig. S1. The total RNA of macrophages was then extracted using a RNeasy Mini Kit (Qiagen). Cyanine-3-labeled cRNA was amplified from 50 ng of total RNA using a Low Input Quick Amp Labeling Kit (Agilent Technologies, Santa Clara, CA, USA). Gene expression profiles were analyzed using SurePrint G3 Mouse GE 8x60K ver.2.0 (Agilent Technologies). Microarray slides were scanned using a Microarray Scanner (Agilent Technologies), and raw fluorescence intensities were quantified and normalized using Feature Extraction software 12.0.3.1 (Agilent Technologies). Raw signal data were processed using Feature Extraction software 12.0.3.1 (Agilent Technologies). Processed data were analyzed using GeneSpring ver. 13.1.1 (Agilent Technologies) or R (R Foundation for Statistical Computing, Vienna, Austria). The microarray data analysis was supported by Hokkaido System Science (Sapporo, Japan). SMG macrophages from eighteen mice or the remaining macrophage subsets from six mice were pooled as one experimental sample, and three independent samples were performed in the microarray analysis.

### Statistical analysis

Experimental values are expressed as means ± SD. Statistical analyses were performed using Prism 7.02 software (GraphPad Software, San Diego, CA, USA) as described in the figure legends. Values of *P* < 0.05 were considered to indicate significance.

## Supplementary Information

Supplementary Information.
