# Supplementary material for: Differential expression of CD11c defines two types of tissue-resident macrophages with different origins in steady-state salivary glands
Source: Sci Rep. 2022 Jan 18;12:931. doi: 10.1038/s41598-022-04941-5 (PMC8766464; doi:10.1038/s41598-022-04941-5)
Supplement: Supplementary file 1 — Supplementary Information. [file 41598_2022_4941_MOESM1_ESM.pdf]

Supplementary Information

Differential expression of CD11c defines two types of tissue-resident macrophages with different origins in steady-state salivary glands

Lu Lu[1], Toshinobu Kuroishi[1], Yukinori Tanaka[2], Mutsumi Furukawa[3], Tomonori Nochi[3], and Shunji Sugawara[1]*

[1]Division of Oral Immunology, Department of Ecological Dentistry, Tohoku University Graduate School of Dentistry, Sendai 980-8575 Japan; [2]Department of Dental Anesthesiology and Pain Management, Tohoku University Hospital, Sendai 980-8574, Japan; [3]International Education and Research Center for Food and Agricultural Immunology, Graduate School of Agricultural Science, Tohoku University, Sendai 980-8572, Japan. *E-mail: shunji.sugawara.d5@tohoku.ac.jp

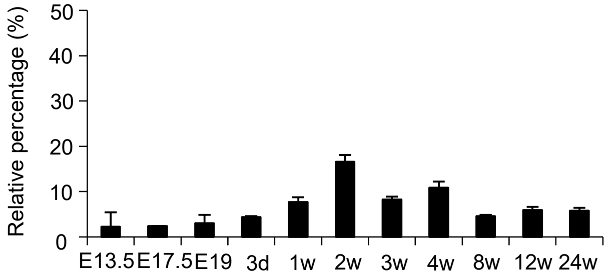

Supplementary Figure S1. Age-dependent transition of cDCs in steady-state SMGs. cDCs in steady-state SMGs from mice at E13.5, E17.5, and E19 (embryo) and at 3 days (newborn) and 1, 2, 3, 4, 8, 12, and 24 weeks after birth were analyzed by flow cytometry. The proportion of cDCs in cDCs and macrophages is shown. Bars represent the mean ± SD (n = 3).

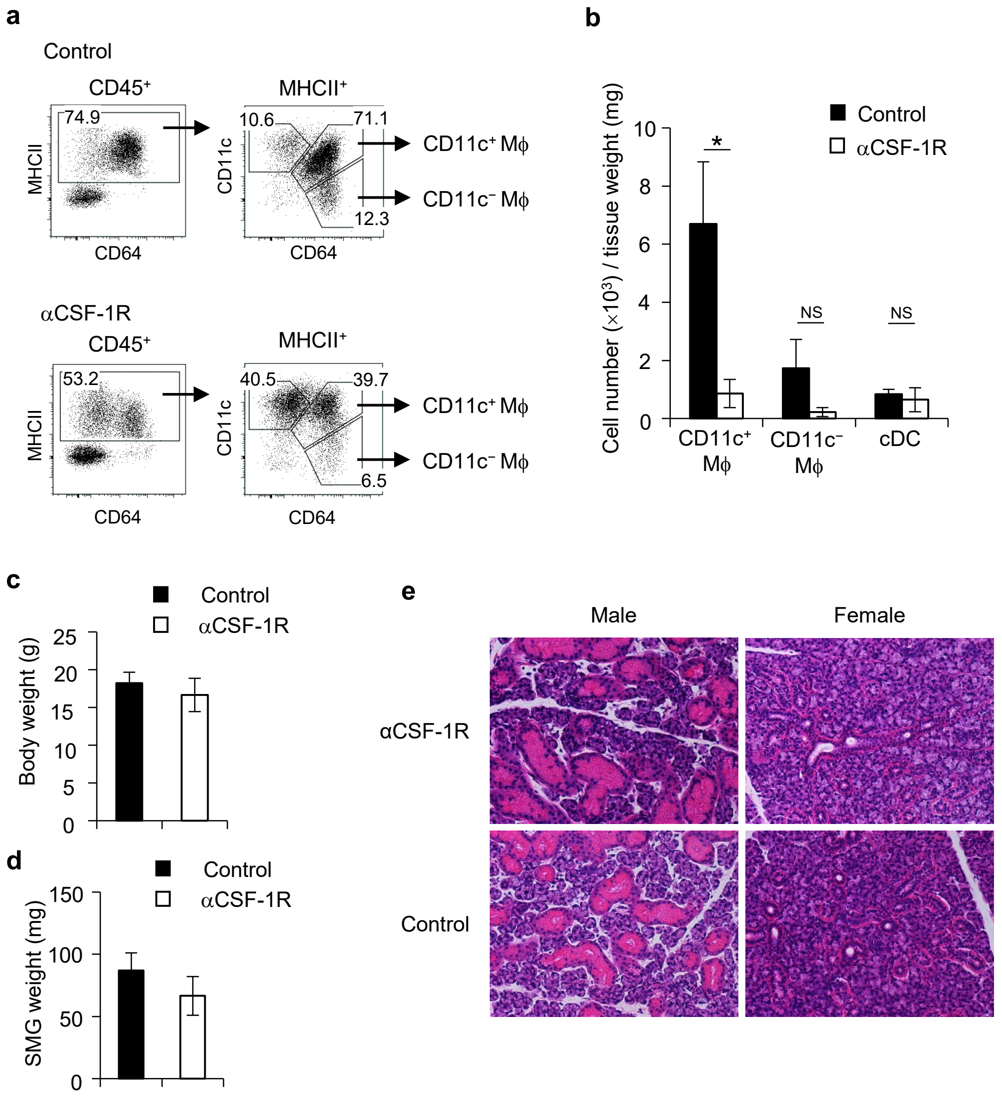

Supplementary Figure S2. Effects of the early-in-life depletion of SG macrophages. One-week-old C57BL/6 mice were intraperitoneally injected with 0.1, 0.025, 0.025, 0.025, and 0.025 mg/g body weight of CSF-1R mAb in 10 μl/g body weight sterile PBS on days 0, 1, 2, 14, and 28, respectively, and then analyzed on day 35. Control mice were injected with PBS. Representative FACS plots (a), the absolute numbers of $CD11c^+$ and $CD11c^-$ macrophages and cDCs (b), body weight (c), SMG weight (d), and hematoxylin-eosin staining of sections in steady-state SMGs from control or anti-CSF-1R-injected mice are shown. Bars represent the mean ± SD (CSF-1R mAb: n = 4, 1 male and 3 female mice. Control: n = 3, 2 male and 1 female mice.). *$P < 0.05$ by the Student's t-test with Welch's correction. Mϕ, macrophage. NS, not significant.

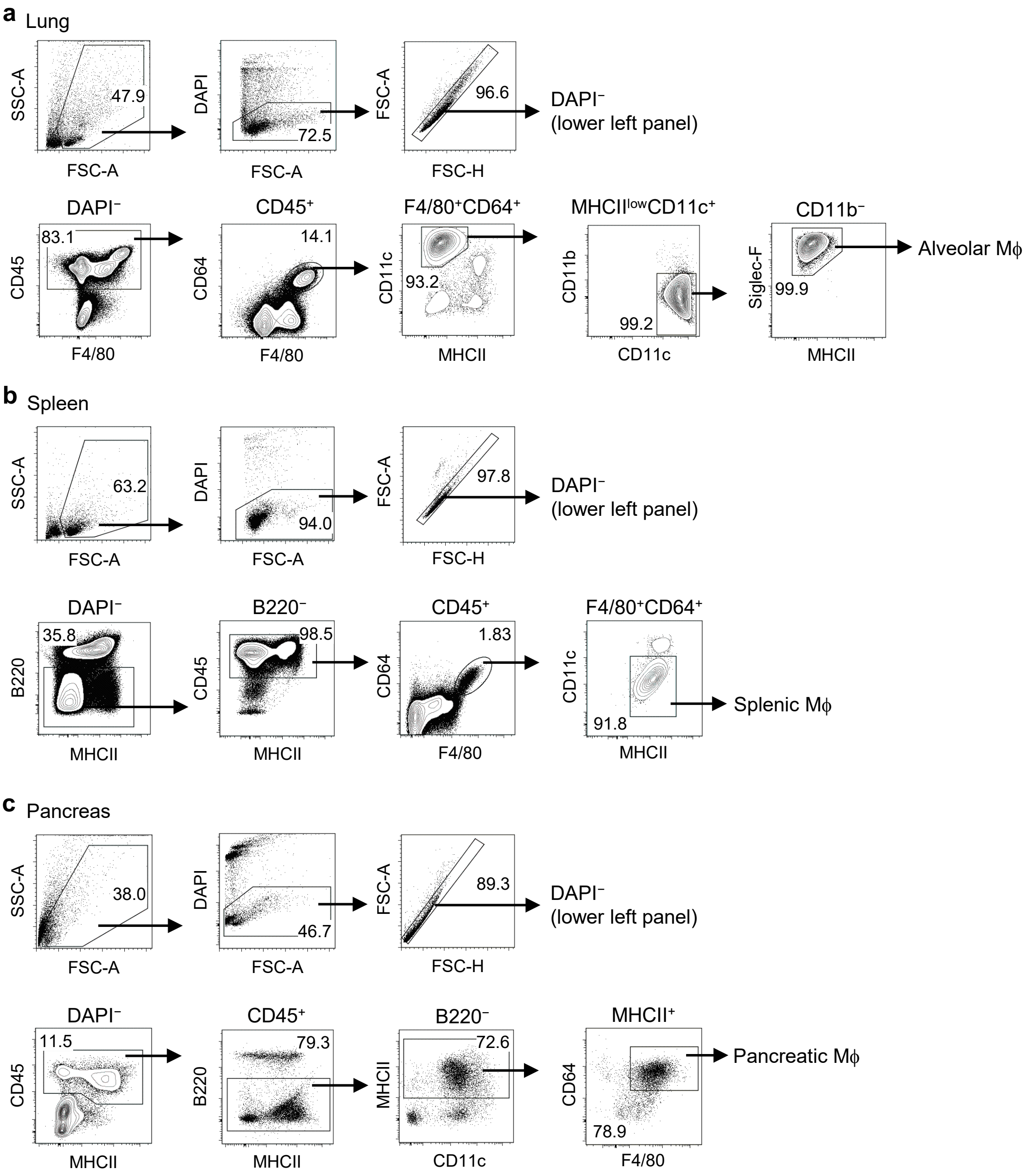

Supplementary Figure S3. Identification of alveolar and splenic macrophages. Gating strategy to identify macrophages in the lung (**a**), spleen (**b**), and exocrine pancreas (**c**). Representative plots are shown. The results are representative of three independent experiments. Mϕ, macrophage.

Supplementary Table S1 Antibodies used for flow cytometry

Antigen detected	Clone	Manufacturer
Annexin V		BioLegend
B220 (CD45R)	RA3-6B2	BioLegend
CD11b	M1/70	BioLegend
CD11c	N418	BioLegend
CD14	Sa14-2	BioLegend
CD45	30-F11	BioLegend
CD45.1	A20	BioLegend
CD45.2	104	BioLegend
CD64	X54-5/7.1	BioLegend
CD206	C068C2	BioLegend
CX3CR1	2A9-1	BioLegend
F4/80	BM8	BioLegend
MHCII (I-A/I-E)	M5/114.15.2	BioLegend
MerTK	Mer	BioLegend
Siglec-F	E50-2440	BD Pharmingen

Supplementary Table S2 Primers used for quantitative RT-PCR

**Primer**	**Sequence**
b-actin Forward	5'-CGTTGACATCCGTAAAGACCTC-3'
b-actin Reverse	5'-AGCCACCGATCCACACAGA-3'
Csf1r Forward	5'-AGGGCCATATACAGGTACACAT-3'
Csf1r Reverse	5'-CACAGGCATCCATGTAACAC-3'
Csf2rb Forward	5'-GGGCAGGAACACAGGACTTCAGGAC-3'
Csf2rb Reverse	5'-CCAGGCCTCTAGCTACCTTGACAGG-3'
Csf1 Forward	5'-TACAAGTGGAAGTGGAGGAGCCAT-3'
Csf1 Reverse	5'-AGTCCTGTGTGCCCAGCATAGAAT-3'
Csf2 Forward	5'-GGCTAAGGTCCTGAGGAGGAT-3'
Csf2 Reverse	5'-ACCTCTTCATTCAACGTGACAGG-3'
Arsg1 Forward	5'-GTAGACCCTGGGGAACACTAT-3'
Arsg1 Reverse	5'-ATCACCTTGCCAATCCCCAG-3'
Fizz1 Forward	5'-CAGCTGATGGTCCCAGTGAAT-3'
Fizz1 Reverse	5'-CAGTGGAGGGATAGTTAGCTGG-3'
Ym1 Forward	5'-GTACCCTGGGTCTCGAGGAA-3'
Ym1 Reverse	5'-CCTTGGAATGTCTTTCTCCACAG-3'
Cd163 Forward	5'-AAAGATGCTGGAGTGACCTG-3'
Cd163 Reverse	5'-GCGTGATCTTTGCTGAAGTTG-3'
Il10 Forward	5'-AGGCGCTGTCATCGATTTC-3'
Il10 Reverse	5'-GACACCTTGGTCTTGGAGCTTA-3'
Il1b Forward	5'-TTCAGGCAGGCAGTATCA-3'
Il1b Reverse	5'-CCAGCAGGTTATCATCATCATC-3'
Arsg Forward	5'-AAAGCCTGTACCGTGCAAGT-3'
Arsg Reverse	5'-CATGGGCCATTGTCTCCTGT-3'
Mertk Forward	5'-ACGTTGGTGGATACGTGCAT-3'
Mertk Reverse	5'-CTCTTCCCACTTCTCGGCAG-3'
Pld3 Forward	5'-CTGATCCTGGCGGTAGTGGG-3'
Pld3 Reverse	5'-CAGACCCTCAGGAATGCTCTC-3'
